# Three-dimensional design, simulation and optimization of a centrifugal compressor impeller with double-splitter blades

**DOI:** 10.1016/j.heliyon.2025.e42011

**Published:** 2025-01-20

**Authors:** Mohammadjavad Tasharrofi, Mojtaba Heidarian Shahri, Ali Madadi

**Affiliations:** Amirkabir University of Technology, Tehran, Iran

**Keywords:** Centrifugal compressor, Double-splitter, Optimization, Artificial network coupled to genetic algorithm, Isentropic efficiency

## Abstract

After the emergence of turbo-machines in the industrial field, researchers have always been seeking to increase their efficiency. Centrifugal compressors are widely used in industries, hence understanding their flow and attempting to increase their efficiency has always been a focus. In recent years, using two splitter blades alongside the main blades has been one of the novel methods used to improve the performance of centrifugal compressors. In this study, after validating the NASA-CC3 centrifugal compressor, a centrifugal compressor design with two splitter blades was carried out. Then, by defining eight variables, an attempt was made to optimize the design. The genetic algorithm was used as the optimization algorithm in this process. Since the optimization process only using genetic algorithms is very time-consuming and has high computational costs, artificial neural networks were used to reduce costs. The objective function in this optimization process was to increase efficiency while maintaining the flow rate and pressure ratio at the design point. After the optimization process was completed, the performance curve of the optimized compressor was compared with the NASA-CC3 compressor at and outside the design point, and its advantages and disadvantages were stated. At the end of the above optimization process, the efficiency of the centrifugal compressor increased by 1.06% at the design point. To investigate the effect of the number of computational networks on the independence of results, further analysis is required.

## Highlights

• The design and optimization of a double-splitter in a centrifugal compressor using computational fluid dynamics coupled with a genetic algorithm.
• Sensitivity analysis of the effect of each design parameter on the performance results of the compressor.
• Presentation of various configurations for modifying the double-splitter design.

**Table:** Nomenclature

*ANN*	*Artificial Neural Network*
*Eqn*	*Equation*
*GA*	*Genetic Algorithm*
*max*	*Maximum*
*min*	*Minimum*
$\dot{m}$	*Mass Flow Rate [kg/s]*
*Imp*	*Impeller*
*PR*	*Pressure Ratio*
*Spl*	*Splitter*
*TR*	*Temperature Ratio*
*V*	*Velocity [m/s]*
*Y*	*Fitness function*
$Y^{+}$	*Y-plus*
Greeks Letter	
η	*Efficiency*
Subscript	
*1*	*First Splitter*
*2*	*Second Splitter*
*C*	*Choke*
*D*	*Design*
*in*	*Inlet*
*NS*	*Near Stall*
*out*	*Outlet*
*T*	*Total*
*0*	*Stagnation property*

## 1 Introduction

Achieving high efficiency in centrifugal compressors while maintaining high pressure ratios requires analyzing their flow physics, identifying complex phenomena within them, and investigating various losses and separations related to centrifugal compressors [1]. One of the recent methods used to reduce blade loading and improve the performance of centrifugal compressors is placing shorter blades between the main blades [2], known as splitter blades. These blades reduce flow separation and increase pressure ratio, preventing flow choking at the inlet of the impeller [3] [4] [5]. In traditional designs, splitter blades have a thickness and angle similar to the main blades and are placed in the middle of the main blades. However, recent studies have focused on optimizing the parameters of this design to improve the efficiency of centrifugal compressors.

With the advancement of computer technology, researchers have been able to design centrifugal compressors with better performance by understanding their internal flow and investigating the phenomena and losses within them. Kang [6] validated the results of a high-pressure ratio centrifugal compressor experiment using computational fluid dynamics and then analyzed the flow. Karrabi et al. [7] compared the numerical results of a centrifugal compressor's performance curve with its experimental results and found good agreement between them. Ibaraki et al. [8] used velocity laser sensors to accurately identify the behavior of the flow in a high-pressure ratio centrifugal compressor. In subsequent years, Ibaraki [9] investigated secondary flows and vortices created by leakage at the blade tip and their interaction with shock waves, leading to significant losses in centrifugal compressors. Numerous other studies have also focused on simulating and analyzing flow in centrifugal compressors and identifying various unknown losses in them [10] [11] [12]. In addition to numerical and experimental studies, analytical studies have also been conducted to predict the performance of centrifugal compressors, such as outlet pressure and efficiency [13].

Recent studies have also investigated the effects of centrifugal compressors with splitter blades on their performance. Fradin [14] conducted extensive experimental studies on two centrifugal compressors, one with a splitter blade and one without. The splitter blade had the same thickness and angle as the main blades and was placed exactly between them. The results showed an improvement in the performance of the compressor with a splitter blade. Studies have shown that when a splitter blade is used, the flow is more uniform when exiting the impeller. Millour [15] demonstrated that the main effect of the splitter blade is to reduce loading on the main blades and thus reduce the jet-wake phenomenon. Oana et al. [16] improved the performance of a centrifugal compressor by changing the thickness of the splitter blade compared to the main blade. Xu and Amano [2] investigated the effects of the location of a splitter blade with specifications similar to the main blade. They considered three locations, exactly in the middle of the two main blades, slightly closer to the pressure surface of the main blade, and slightly closer to the suction surface of the main blade, and examined the results. They showed that the conventional design is not the optimal configuration and that the performance of the compressor improves as the splitter blade approaches the pressure surface.

Optimizations of turbomachines have been studied by many researchers. In 2004, Benini [17] worked on the optimization of NASA Rotor-37. He did this optimization with the two maximum goals of increasing isentropic efficiency and pressure ratio by restricting the mass flow rate. Finally, by keeping the pressure ratio and mass flow constant, he was able to increase the isentropic efficiency by 1.5%. Also, he was able to increase the pressure ratio up to 5.5% by reducing the isentropic efficiency by 0.8%. Heidarian et al. [18] and Sarabchi et al. [19] optimized the lean and sweep of NASA rotor 67 and led to a 0.57% increase in isentropic efficiency and a 0.93% increase in pressure ratio. In this optimization, they were helped by coupling genetic algorithm and neural network. Also, in two another article, Heidarian et al. optimized the squealer tip of an axial compressor (NASA Rotor-67) [20] and a centrifugal compressor (NASA-CC3) [21], which led to a 4.81% and 2.14% increases in surge margin, respectively. In this research, they used the design of experiments using the Taguchi algorithm method to measure the sensitivity of the parameters and formed their own database, and then used the coupling of the genetic algorithm and the neural network to accelerate the optimization process. Ekradi and Madadi [22] used an artificial neural network and a genetic algorithm to optimize the distribution of angles between the main and splitter blades of a transonic centrifugal compressor, improving its performance at the design point and outside it. Ju et al. [23] optimized the hub and shroud using a genetic algorithm and artificial neural network, making the flow exiting the impeller more uniform at the design point and increasing efficiency in the compressor's operating range. Xiao et al. [24] improved the efficiency of a transonic centrifugal compressor by optimizing the curvature and sweep of its blades using a combination of genetic algorithm and artificial neural network, achieving a 2.2% improvement in efficiency. Moussavi et al. [25] investigated the effects of the location and angles of the splitter blade's leading edge on a centrifugal compressor's performance, resulting in a 2.7% improvement in efficiency.

Tamaki et al. [26] used two splitter blades as one way to increase pressure ratio in centrifugal compressors and reduce loading on the main blades. Higashimori et al. [27] investigated and understood the flow in a transonic centrifugal compressor with a Mach number of 1.6 and a pressure ratio of 11 used for aviation applications. Marconcini et al. [28] studied phenomena such as blade tip leakage, shock waves, secondary flows, and their interactions at the design point and outside it in a transonic centrifugal compressor to better understand the physics of flow. Malik and Zheng [29] used two splitter blades to increase pressure ratio from 4.1 to 4.4 and improve efficiency by 2%.In another study, they also improved the performance and pressure ratio of a centrifugal compressor by converting it from a single splitter blade to two splitter blades. They also presented two designs of centrifugal compressors with two splitter blades. In the first configuration, the first splitter blade is close to the suction surface and the second splitter blade is close to the pressure surface, while in the second configuration, the first splitter blade is close to the pressure surface and the second splitter blade is close to the suction surface. They showed that in the second configuration, the flow exiting the impeller is more uniform, and the compressor exhibits better efficiency and performance.

Optimization of two splitter blades is considered as the objective of this article which have not been investigated by previous researchers. The parameters of the double-splitter have significant effects on the compressor's performance. The design and relative placement of the second splitter blade (placed between the main blade and the first splitter blade for example) and its size, whether shorter or larger than the first splitter blade, have all been selected as initiatives in the optimization objectives of this problem. The optimized centrifugal compressor with the second splitter blade has resulted in reduced flow losses, increased efficiency, increased compressor pressure ratio, and improved surge margin, as described and elaborated in the results of the characteristic curve, blade loading, circumferential and radial distribution of performance parameters, and the surge margin.

## 2 NASA-CC3 centrifugal compressor

The computational fluid dynamics solution algorithm is utilized for the three-dimensional geometry of the NASA-CC3 centrifugal compressor, as mentioned in the aforementioned configuration.

### 2.1 Three-dimensional geometry

**Fig. 1:**
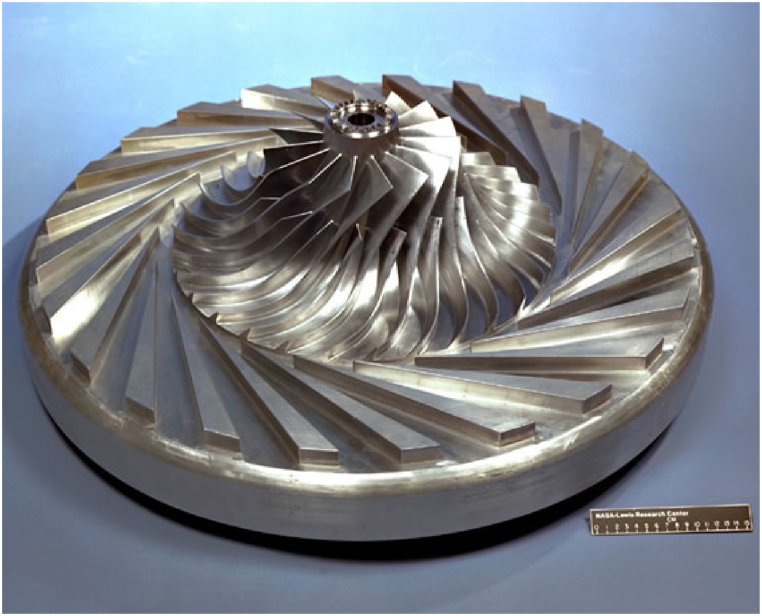
Three-dimensional geometry of NASA-CC3 centrifugal compressor.

The design of the NASA-CC3 centrifugal compressor, in 1975, was carried out for use in gas turbines with heat recovery systems used in transportation or power plants. This compressor has 15 main blades and 15 splitter blades. The general specifications of this compressor are presented in Table 1. The impeller beta angle and thickness data used to create the geometry in this study are extracted from reference [1]. The three-dimensional picture of this compressor is shown in Fig. 1.

**Table 1:** Design point and geometrical data of the compressor.

Parameter	Unit	Value
Pressure ratio	[]	4
Rotational Speed	[rpm]	21789
Impeller tip speed	[m/s]	492
No. of main blades	[]	15
No. of splitter blades	[]	15
No. of diffuser blades	[]	24
Impeller exit diameter	[m]	0.43
Tip clearance at leading edge	[mm]	0.1524
Tip clearance at trailing Edge	[mm]	0.2030
Backswept from radial	[deg]	50

**Fig. 2:**
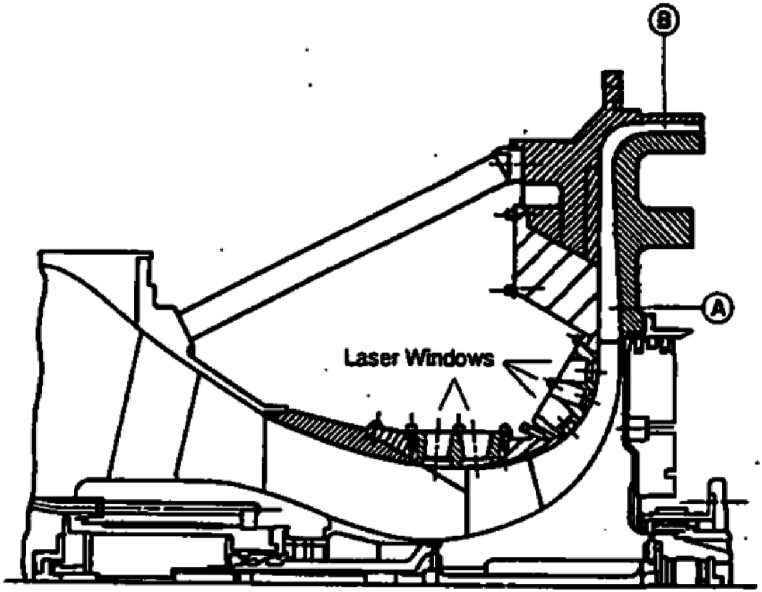
The meridional view of NASA-CC3 centrifugal compressor [30].

Testing of this compressor was carried out at Allison Engine Company using laser anemometry to obtain fluid flow velocity. The meridional view is for the aforementioned compressor, and further aerodynamic performance data and comparison with numerical results are depicted in Fig. 2.

### 2.2 Grid generation

**Fig. 3:**
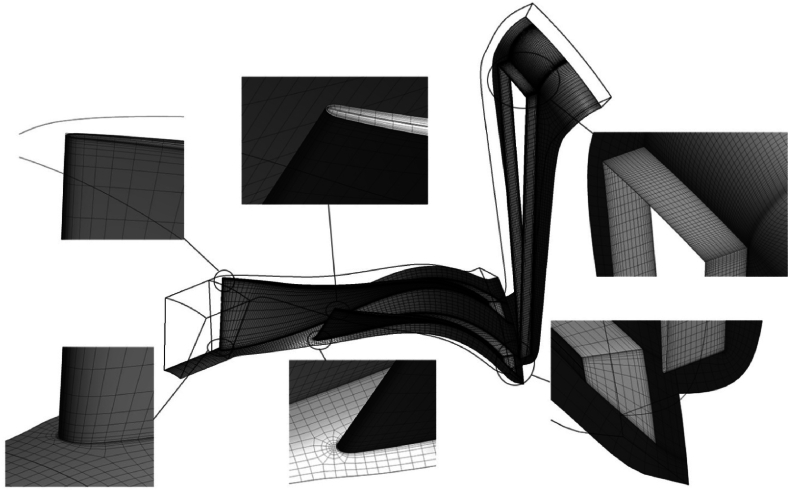
The medium size structured grid of NASA-CC3 centrifugal compressor.

To generate a structured computational grid and analyze the aerodynamic flow, an impeller, a splitter, and a diffuser were used to reduce computational costs. Therefore, periodic conditions can be used to solve the problem. Accuracy in validating the boundary layer grid is crucial to ensure capturing the boundary layer effects in this analysis is of paramount importance. Fig. 3 show the grids generated for this centrifugal compressor.

### 2.3 Boundary conditions

**Fig. 4:**
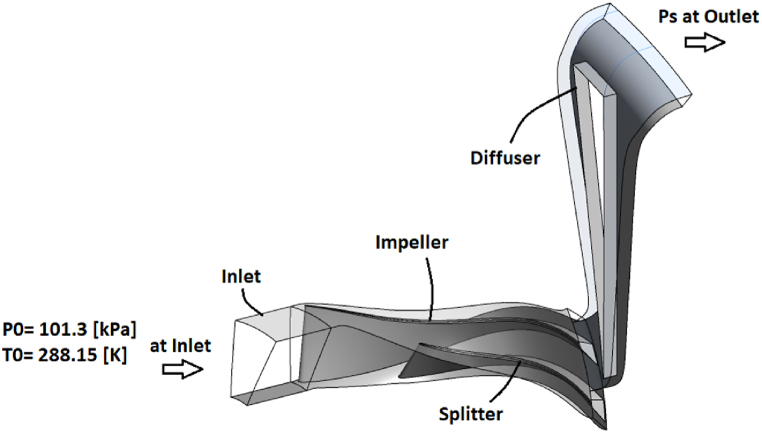
The boundary condition used in numerical simulation of NASA-CC3 centrifugal compressor.

The flow field is solved in a steady manner based on the Reynolds-averaged Navier-Stokes equations. In this solution, the Shear Stress Transport turbulence model, which is one of the most reliable turbulence models for solving flow fields in the field of turbo-machinery, is used. For all walls, the boundary condition of no-slip and adiabatic heat transfer is considered. At the inlet, total pressure and temperature conditions are used with values of 1 atm and 288.15 K, and at the outlet, the boundary condition of static pressure is used as t is depicted in Fig. 4.

### 2.4 The grid study investigation

One of the influential factors on the results of numerical solution is the number of elements in the computational grid. To this end, 4 computational grids with different numbers of cells have been considered, and variations in the mass flow rate and efficiency values have been taken into account to select the appropriate grid. The number of elements for each grid is shown in Table 2.

**Table 2:** The grid elements of impeller and diffuser for grid study.

Row	Impeller Elements	Diffuser Elements	Total Elements
1	200,000	130,000	330,000
2	430,000	250,000	680,000
3	800,000	500,000	1,300,000
4	1,400,000	740,000	2,140,000

**Fig. 5:**
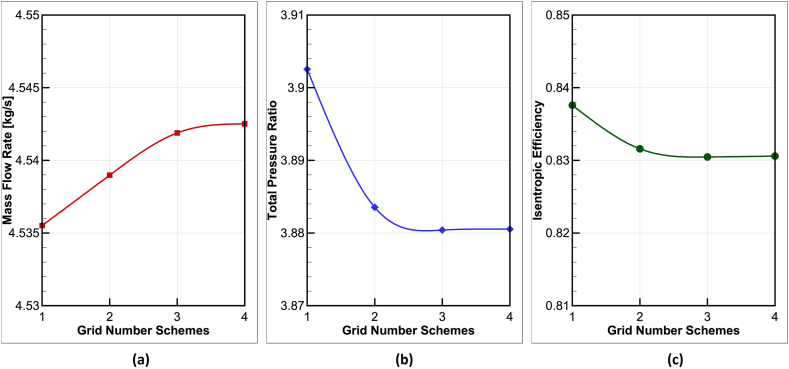
The grid study used in the numerical simulation of the NASA-CC3 centrifugal compressor (a) mass flow rate, (b) total pressure ratio, (c) isentropic efficiency.

Fig. 5 respectively illustrate the variations in mass flow rate, efficiency, and pressure ratio as a function of the number of cells in the computational grids. It is also worth mentioning that in order to check the independence of the network, due to better convergence, the outlet static pressure is considered at the design point of the compressor. The value of this static pressure is 370 kPa.

Based on the obtained results, convergence of the parameter values is observed in the fourth grid; therefore, the fourth grid has been used for further analysis.

**Fig. 6:**
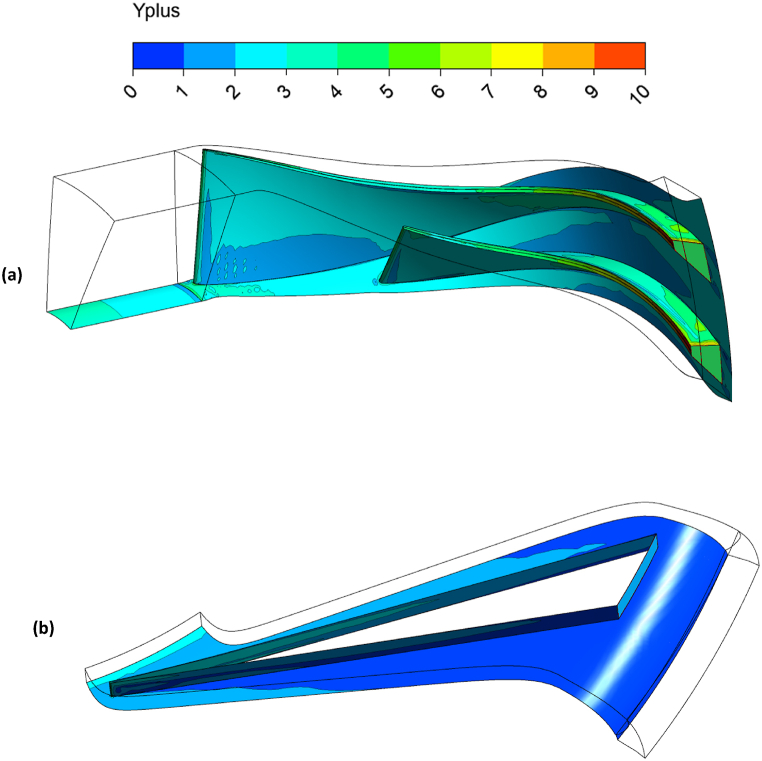
The Y-plus distribution of grid no.4 in numerical simulation for (a) impeller and splitter, and (b) diffuser.

To assess the accuracy of the SST turbulence model in the fourth grid, the distribution of the y-plus is presented in Fig. 6, which should be in the permissible range of 1–5. The distribution in the impeller and splitter regions reaches approximately 4–5 in Fig. 6, while it averages between 1 and 2 in the diffuser. Thus, these results can confirm the accuracy employed for the fourth grid. Previous studies conducted by the authors on similar test cases have also confirmed that the selected grid adheres to the permissible y-plus range in 90% of the regions [20].

### 2.5 CFD solver validation

At the end of this section, the results of the numerical solution have been compared with the available experimental results for the NASA-CC3 radial compressor. As mentioned in reference [1], experimental data is available in two cases, with and without a diffuser, and in the current study, a diffuser has been used after the compressor impeller.

**Fig. 7:**
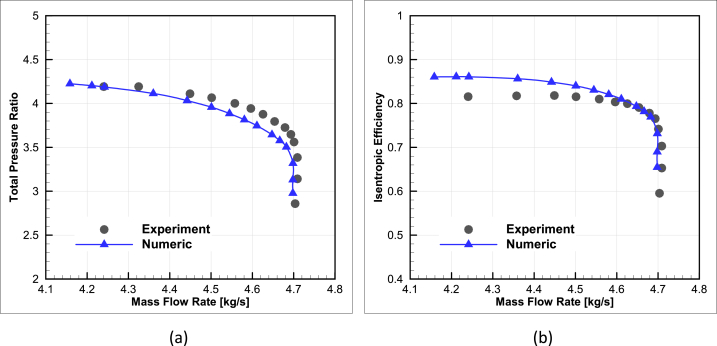
The performance map validation of NASA-CC3 centrifugal compressor for (a) mass flow rate vs. total pressure ratio and (b) mass flow rate vs. isentropic efficiency.

Considering the differences between the numerical and experimental results at the compressor design point, it can be stated that the errors are within an acceptable range. At the design point, the efficiency error is 0.17%, the mass flow rate error is less than 0.06%, and the pressure ratio error is 2.25%. Additionally, performance curves have been compared with the experimental results based on the obtained data. Fig. 7 show the efficiency and pressure ratio versus mass flow rate.

## 3 The optimization process

This section discusses the creation of a compressor impeller with two splitter blades and introduces the optimization variables in this research. It then explains the development of the optimization code, the application of variables, the design of experiments using the Taguchi method, and the formation of a database, sensitivity analysis of objective functions to design variables, creation of neural networks, validation, and genetic algorithm are presented.

### 3.1 Design variables and objective functions

In this research, the specifications and configuration of the main impeller are used to create new splitter blades. In other words, the thickness distribution and angles of the main impeller do not differ from the first and second splitter blades. Furthermore, the hub and shroud curves and the diffuser have not changed compared to the NASA-CC3 compressor.

Another condition that will be further explained in detail is the need for the second splitter blade to be smaller than the first splitter blade. Also, the number of blades should be minimized to achieve the objective of minimizing mass flow loss, which is one of the optimization variables. So, N refers to the number of segments made by a main blade, a first splitter, and a second splitter.

Another important point is the existence of two configurations for radial compressors with two splitter blades. In the first configuration, the larger splitter blade is closer to the pressure surface of the main impeller, and the smaller splitter blade is closer to the suction surface of the main impeller. In the second configuration of radial compressors with two splitter blades, the larger splitter blade is closer to the suction surface of the main impeller, and the smaller splitter blade is closer to the pressure surface of the main impeller.

**Fig. 8:**
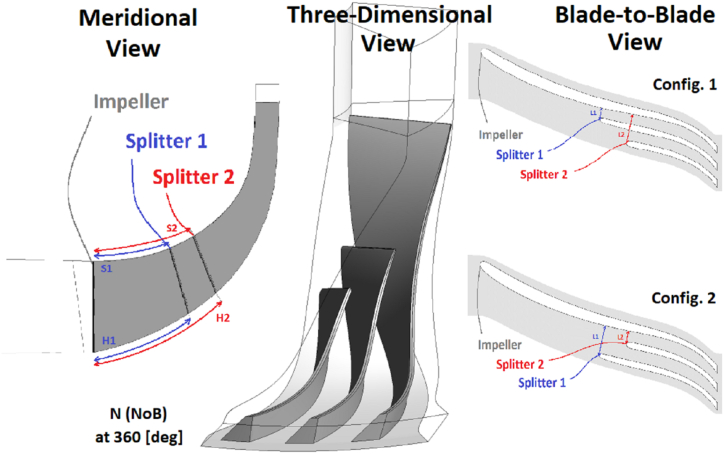
The parameterization of centrifugal compressor for design variable.

The starting point of the first and second splitters in the meridional view, as well as the distances of the splitters relative to the impeller, along with the number of blades, are parameters of the design shown in Fig. 8. It should be noted that the parameters S and H are defined relatively from inlet to outlet which means that the inlet corresponds to 0 and outlet corresponds to 1. Also, L is defined relatively from suction side to pressure side of the main blade in the blade-to-blade view. The default relative locations of the splitter blades are 0.333 and 0.6667. To calculate L1 and L2, two numbers between −0.05 and 0.05 are considered, which are added to the numbers 0.333 and 0.667 based on the configuration. In the first configuration, L1 is added with 0.333 and L2 with 0.667. In the second configuration, L1 will be added with 0.667 and L2 with 0.333. The upper and lower limits for this problem are mentioned in Table 3.

**Table 3:** The upper and lower parameters.

Design Variables	Configuration	N	H1	H2	S1	S2	L1	L2
Maximum	1	12	0.85	0.85	0.85	0.85	+0.05	+0.05
Minimum	2	10	0.2	0.2	0.2	0.2	−0.05	−0.05

According to the definition of creating double splitters, if the order of the blades is impeller, first splitter, and second splitter, it is called configuration 1; and if the order is impeller, second splitter, and first splitter, it is called configuration 2.

The objective functions of the problem include performance parameters such as total pressure ratio, isentropic efficiency, and mass flow rate at the compressor design point, in which the inlet value is evaluated under atmospheric conditions.

### 3.2 Design of experiment and sensitivity analysis

A design and optimization of a centrifugal compressor with the addition of a second splitter has been carried out using artificial intelligence based on pre-validated numerical data. Therefore, the production of a database and sensitivity analysis of the impact of each design parameter on the objective functions were the goals of this study. One of the methods used to form a database and parameter sensitivity is the Taguchi algorithm, which Heidarian et al. used in Ref [20] [21]. So for each of these two installed splitter configurations, a Taguchi experimental design was written. Considering the number of design variables and their three levels of variation, an L-27 orthogonal array Taguchi design was selected according to Table 4. In total, two datasets for two configurations of the splitter blades have been prepared (54 simulations), to compute the sensitivity of performance parameters to the design variables.

**Table 4:** The levels of variables in Taguchi L-27 orthogonal arrays.

Factor Experiments	1	2	3	4	5	6	7	8	9	10	11	12	13	14	15	16	17	18	19	20	21	22	23	24	25	26	27
N	1	1	1	1	1	1	1	1	1	2	2	2	2	2	2	2	2	2	3	3	3	3	3	3	3	3	3
H1	1	1	1	2	2	2	3	3	3	1	1	1	2	2	2	3	3	3	1	1	1	2	2	2	3	3	3
H2	1	1	1	2	2	2	3	3	3	2	2	2	3	3	3	1	1	1	3	3	3	1	1	1	2	2	2
S1	1	1	1	2	2	2	3	3	3	3	3	3	1	1	1	2	2	2	2	2	2	3	3	3	1	1	1
S2	1	2	3	1	2	3	1	2	3	1	2	3	1	2	3	1	2	3	1	2	3	1	2	3	1	2	3
L1	1	2	3	1	2	3	1	2	3	2	3	1	2	3	1	2	3	1	3	1	2	3	1	2	3	1	2
L2	1	2	3	1	2	3	1	2	3	3	1	2	3	1	2	3	1	2	2	3	1	2	3	1	2	3	1

**Fig. 9:**
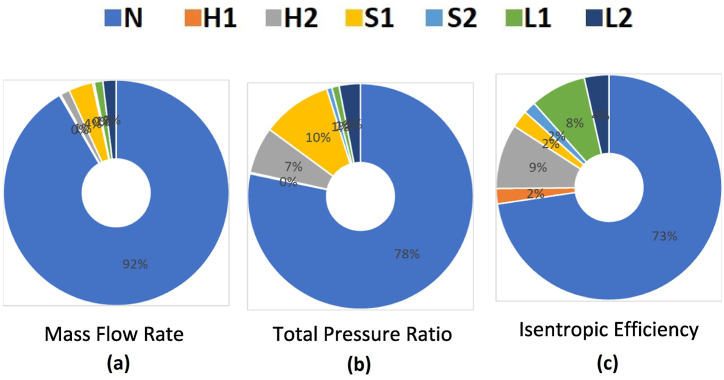
The sensitivity analysis of design variables for the new centrifugal compressor with a double-splitter (a) mass flow rate, (b) total pressure ratio, (c) isentropic efficiency.

According to the research by Heidarian et al. [20] [21], for sensitivity analysis of design parameters in the study of producing a squealer-tip centrifugal compressor, the sensitivity effect (positive or negative) of each of the performance parameters of this design was obtained using variance from the results of the performance parameters of the centrifugal compressor (pressure ratio, mass flow rate, and isentropic efficiency), which can be seen in Fig. 9 for each of the configurations.

The results show that:

1 It has been inferred that the number of blades (N) plays an important role in all three performance parameters as explained by the mass flow rate, total pressure ratio, and isentropic efficiency.
2 The start location of the first splitter on the shroud (S1) and the start location of the second splitter on the hub (H2) have the next effective parameters in total pressure ratio.
3 It appears that changes in the placement lengths of splitters relative to the impeller may have an impact on isentropic efficiency. However, it is insignificant in terms of the total pressure ratio.

### 3.3 Artificial neural network coupled to genetic algorithm

**Fig. 10:**
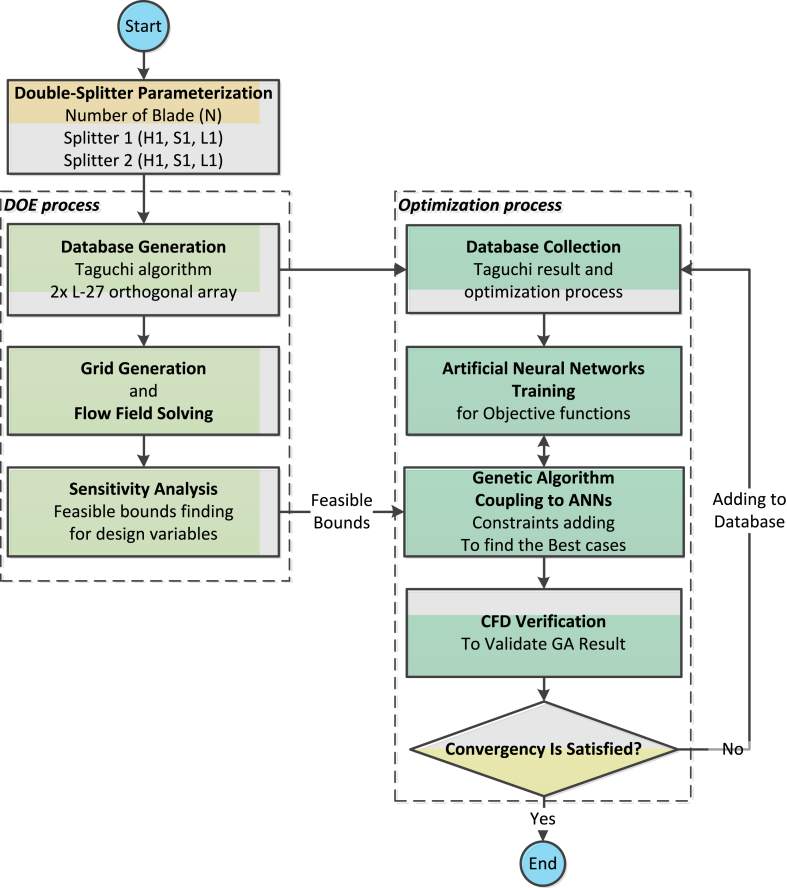
Optimization process of centrifugal compressor.

The use of numerical optimization methods coupled with computational fluid dynamics is a common approach in the search for optimal points and achieving improved performance. This coupling has been elucidated in the study of three-dimensional optimization of axial compressors by Benini [17], and developed by Heidarian et al. [18] [20]and Ekradi et al. [22] (Fig. 10).

The objective function that is defined is in accordance with the stated goal, i.e. in such a way that the efficiency increases by maintaining the mass flow rate and pressure ratio. For this reason, it is specified in the objective function that the mass flow rate should not be less than 4.54 kg/s and the pressure ratio should not be less than 3.88 and the efficiency should be increased. The objective function formula is shown in Eqn. (1).

(1) $$Y=-1*\eta +\max\left(0,\left(4.54-\dot{m}\right)\right)+\max\left(0,\left(3.88-\mathrm{PR}\right)\right)$$

The penalty function technique (that has been used before by the authors at [18] [20] [22]) is employed for constraining the design mass flow rate and design pressure ratio higher than the original impeller. If the mass flow rate and pressure ratio are greater than the original impeller, the penalty function corresponds to zero. Otherwise, a penalty is added to the objective function and the genetic algorithm tries to reduce it.

#### 3.3.1 Validation and regression

**Fig. 11:**
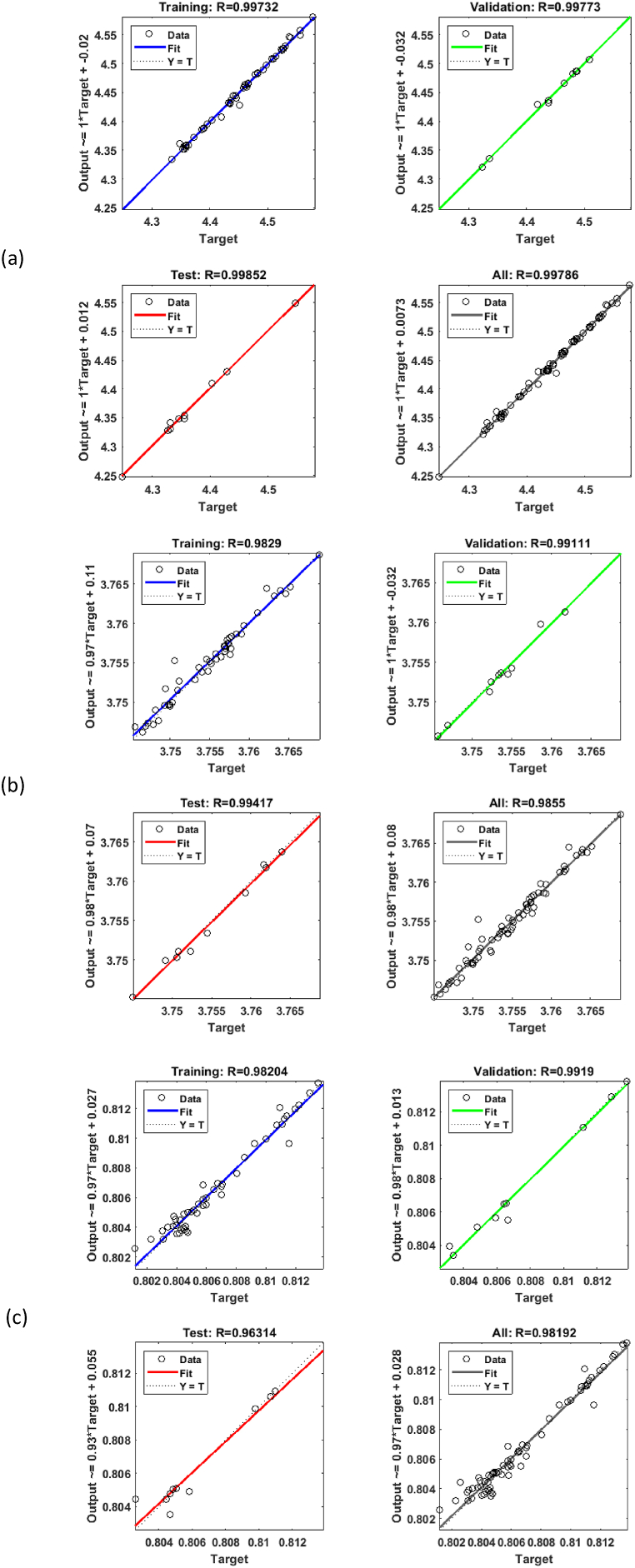
Artificial neural networks validation and regression for the (a) Mass flow rate, (b) Total pressure ratio, and (c) Isentropic efficiency at the design point.

In this research, inspired by the aforementioned studies, as shown in Fig. 10, an initial database (in this case Taguchi algorithm studies) is used to train artificial neural networks in the optimization process. The three neural networks are used for the objective functions of pressure ratio, mass flow rate, and isentropic efficiency, and the trained artificial neural networks, along with their regression (which demonstrates the high accuracy of the networks), are illustrated for the specified objective functions in Fig. 11. Results indicate that a validation accuracy of 99% has been successfully achieved by the networks. The ANN settings are presented in Table 5.

**Table 5:** The artificial neural network settings.

Criteria	Function/Value
Number of layers	3
Number of neurons	5
Feeding method	Back Propagation
Number of epochs	500
Convergence goal	1e-16
Output function	Liner Function
Neuron activation function	Sigmoid
Data division	Random
Training algorithm	Levenberg-Marquardt
Performance	Mean Squared Error

**Fig. 12:**
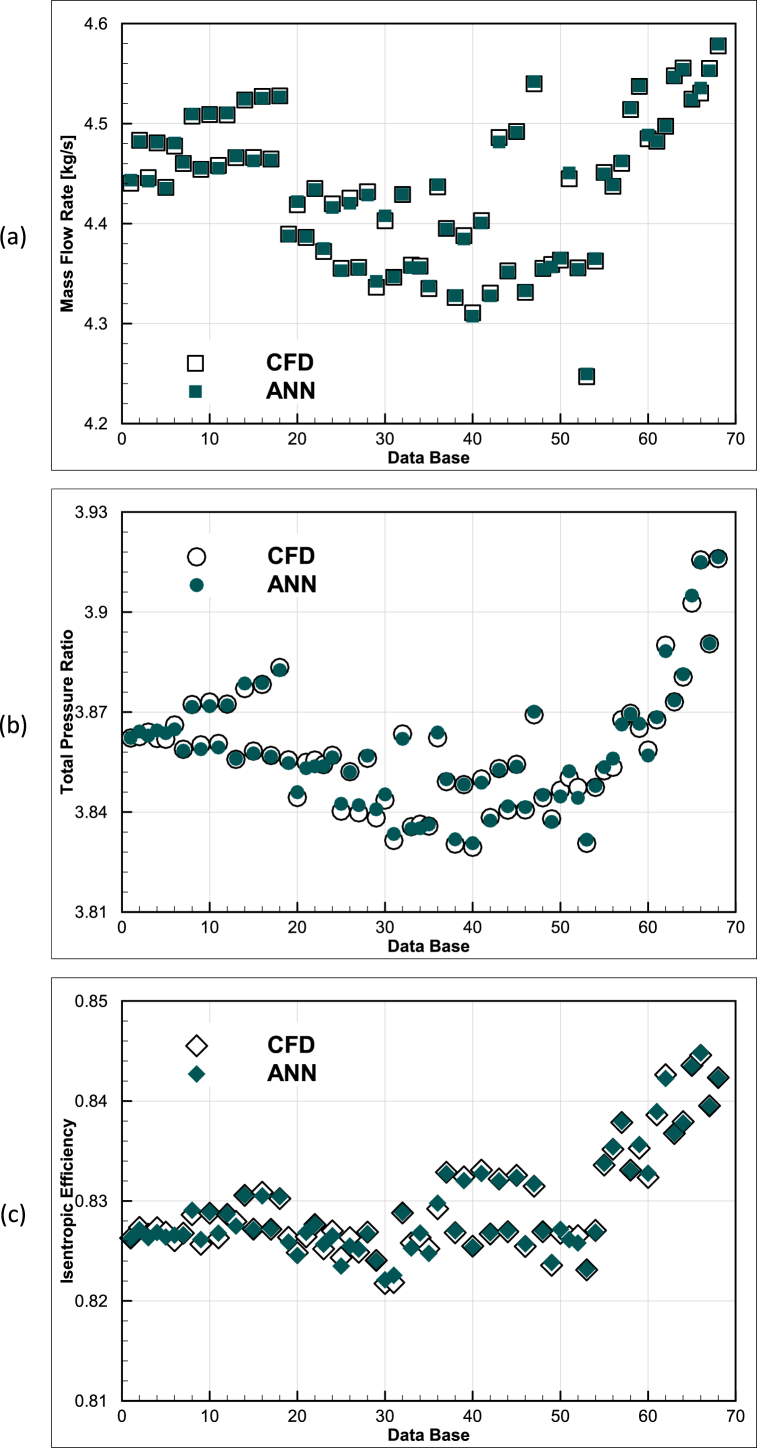
Comparison of the CFD results and artificial neural networks (a) Mass flow rate, (b) Total pressure ratio, and (c) Isentropic efficiency at design point.

For comparison, the results of the networks can also be presented alongside the CFD data as shown in Fig. 12, which corroborates the aforementioned 99% validation accuracy.

#### 3.3.2 Genetic algorithm

A genetic algorithm optimization is applied to the artificial neural networks, and the search for optimal points is conducted. The advantage of this coupling of artificial neural networks with genetic algorithms is the reduction in computational costs. The optimized points in each coupling are also validated using CFD, and their convergence accuracy is examined. If the obtained results do not have sufficient accuracy, the initial database is updated by adding the new CFD results to increase the accuracy, and the networks are retrained. In cases where the optimization results have acceptable accuracy, the optimization loop is exited, and the optimized compressor with double-splitter is presented.

**Fig. 13:**
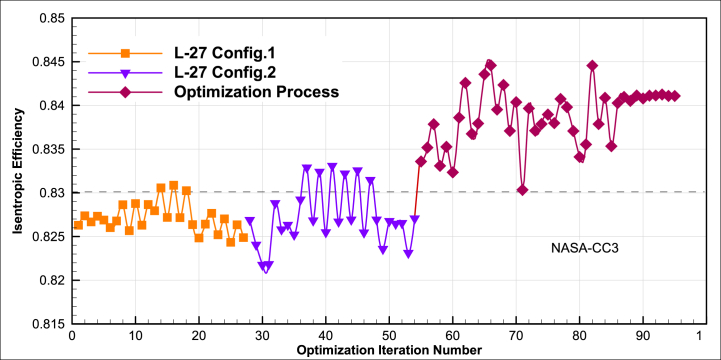
The convergence of design isentropic efficiency at the optimization problem.

The convergence of isentropic efficiency of this optimization procedure is shown in Fig. 13.

**Fig. 14:**
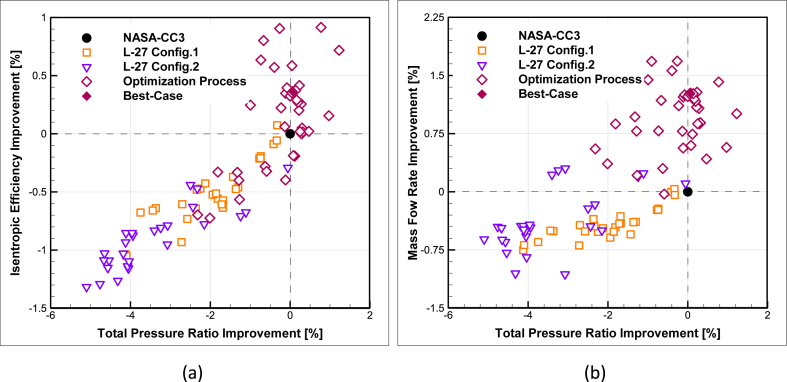
The percentage improvement of database and optimization algorithm compared to NASA-CC3 for (a) Total pressure ratio-isentropic efficiency and (b) Total pressure ratio-Mass flow rate.

The variation trends of the performance parameters of total pressure ratio, mass flow rate, and isentropic efficiency, compared to the design point results of the NASA-CC3 centrifugal compressor, in three steps of Taguchi orthogonal array database for configuration 1, configuration 2, and numerical optimization process, are depicted in Fig. 14.

## 4 Results and discussion

The innovative philosophy behind the double splitter design in centrifugal compressors is derived from the splitter design philosophy aimed to enhance the performance of centrifugal compressors. The variation in cross-sectional area from the inlet to the outlet of a centrifugal compressor is significant; therefore, in the absence of a splitter, flow separation occurs at the outlet. If the number of impellers is increased from the leading edge, a blockage occurs at the inlet of the centrifugal compressor, leading to an increase in flow Mach number. For this reason, the geometry of the splitter has been designed so that air begins to flow at the compressor inlet (where the area is small) with a lower number of blades. By increasing flow density through the flow path, a splitter blade is introduced to divide the flow between two channels, the impeller, and the splitter. Thus, based on the aforementioned explanations and to improve the effect of the splitter, the concept of the double-splitter can be articulated. This allows to increase the number of blades in two steps which results in a gradual change of the area and number of flow channels from inlet to outlet.

In this section, the geometric characteristics of the optimized impeller and then the performance curve of this impeller in comparison to the baseline geometry have been presented. Finally, the advantages, including the improved performance of this impeller, blade loading, and circumferential and radial performance distributions are discussed.

### 4.1 Geometry specification

**Fig. 15:**
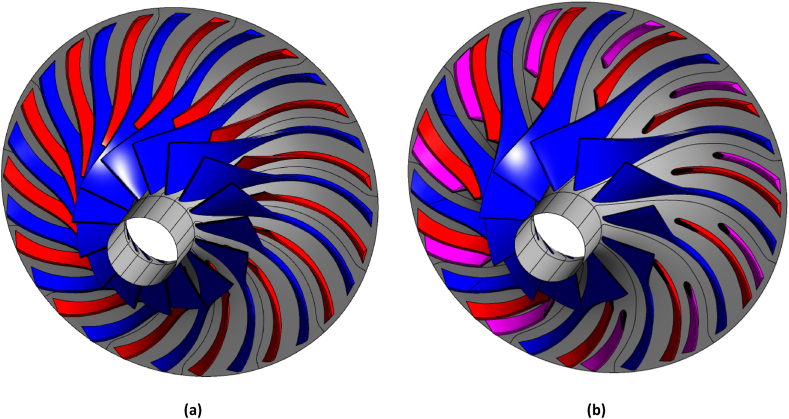
The comparison of three-dimensional geometry for (a) NASA-CC3 and (b) optimal centrifugal compressor.

The optimized impeller has eleven main blades, first and second splitters. This impeller has the configuration of the larger splitter blade closer to the pressure side of the main blade and the smaller splitter blade closer to the suction side of the main blade (configuration 1). The geometric characteristics of the splitters blades are similar to the main blade, which is derived from the NASA-CC3 impeller. Additionally, there are no changes in the hub and shroud profiles of the optimized impeller compared to the NASA-CC3 impeller. Fig. 15 shows the three-dimensional geometry of the base-line compressor compared to best-case ones.

The optimized compressor geometrical parameters are presented in Table 6.

**Table 6:** Geometrical parameters of NASA-CC3 and optimized compressor.

Design Variable	Configuration	N	H1	H2	S1	S2	L1	L2
NASA-CC3	–	15	0.3	–	0.3	–	0.5	–
Best-Case	1	11	0.5	0.714	0.5	0.714	0.6167	0.3833

Another result of this optimization is the reduction in the weight of the impeller, which is particularly important in rotor-dynamic performance. Table 7 presents the description of changes in fluid volume and structure (impeller and splitter) for two baseline and optimal geometries.

**Table 7:** Geometrical parameters of NASA-CC3 and optimized compressor.

Domain	Structure		Fluid	
	Volume [mm^3^]	Normalized Change [%]	Volume [mm^3^]	Normalized Change [%]
NASA-CC3	718371	–	4629540	–
Best-Case	349654	−51.32	4998257	+7.96

Assuming that the material of the NASA-CC3 compressor and the optimized compressor is the same [11], The density of both compressors are identical. Therefore, considering the 51.32% normalized reduction in the volume of the blades in the optimized compressor, the mass of the blades in the optimized compressor is reduce, too. It should be noted that this reduction in mass is only related to the mass of the blades, including the main blade, the first splitter blade, and the second splitter blade. The mass of the compressor also includes the hub, which constitutes the main portion of the weight.

### 4.2 Performance characteristic maps

**Fig. 16:**
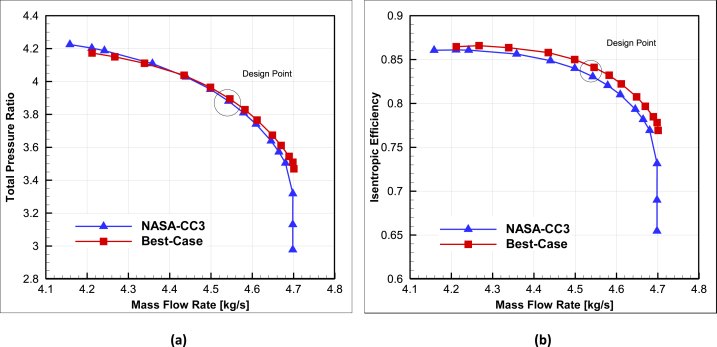
The comparison of optimal centrifugal compressor and NASA-CC3for (a) total pressure ratio-mass flow rate and (b) isentropic efficiency-mass flow rate.

The first step in examining and displaying the improved performance of the optimized compressor compared to the baseline is to compare the efficiency performance curve in terms of flow rate and pressure ratio. In Fig. 16, the efficiency and total pressure ratio versus mass flow rate curve of the baseline compressor is compared with the optimized compressor.

As it is evident, the optimized compressor performs better than the baseline compressor in all operating conditions, with a higher efficiency. This increase in efficiency at the design point is approximately 1.06%, and it varies with movement towards choke. Another notable point is that there is no difference in choke mass flow rate of best-case in comparison with baseline (approximately 4.7 kg/s).

Based on the curve, it can be said that throughout the operating conditions of the compressors, the differences in total pressure ratio between them are very small. As the optimized compressor approaches choke points, it provides slightly higher total pressure ratio, but as it approaches surge points, its total pressure ratio decreases slightly compared to the baseline compressor.

### 4.3 Loading distribution

#### 4.3.1 Impeller loading

**Fig. 17:**
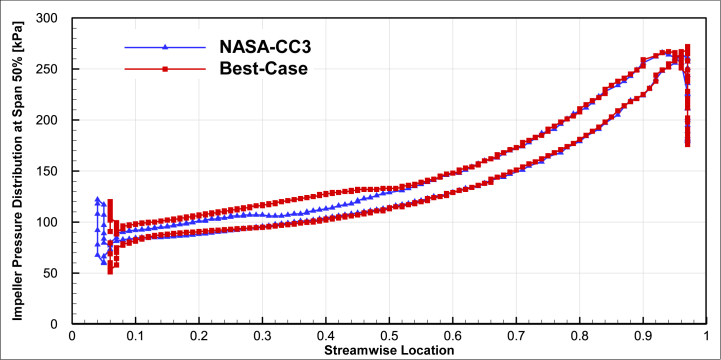
The comparison of impeller pressure distribution of the optimal compressor and NASA-CC3 at span 50%.

**Fig. 18:**
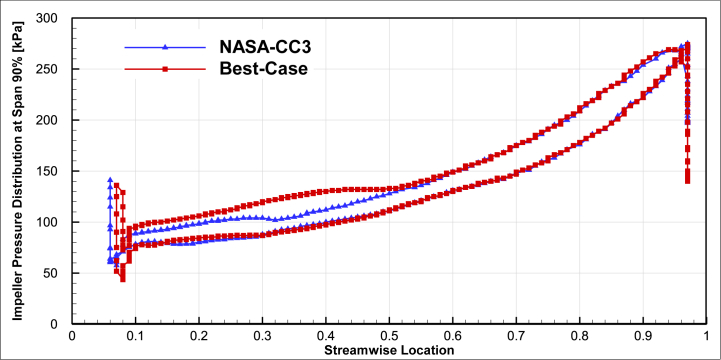
The comparison of impeller pressure distribution of the optimal compressor and NASA-CC3 at span 90%.

Another result of the improved compressor optimization compared to the baseline compressor is the increase in static pressure at the first part of the impeller as shown in Fig. 17 for 50% span and Fig. 18 for 90% span. Because of reduction in the number of blades, in this section only the main blade and the first splitter exist in the flow field and the cross-sectional area is increased and the velocity is decreased. Velocity reduction, results in static pressure increment.

#### 4.3.2 Splitter loading

**Fig. 19:**
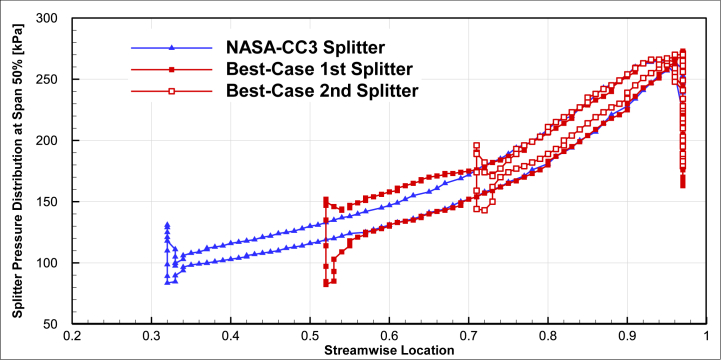
The comparison of splitter pressure distribution of the optimal compressor and NASA-CC3 at span 50%.

**Fig. 20:**
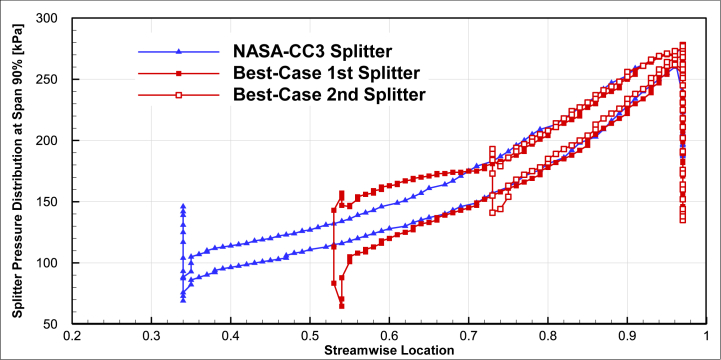
The comparison of splitter pressure distribution of the optimal compressor and NASA-CC3 at span 90%.

A comparison of the pressure distribution on the surface of optimized splitter blades was performed in comparison to the baseline blade geometry for 50% span in Figs. 19 and 90% span in Fig. 20. The chord lengths of both optimized splitters were smaller than that of the baseline splitter, but the pressure distribution on the surface of both optimized splitters was greater than that of the baseline splitter. An increase in surface pressure at the leading edge of the first splitter (around streamwise location 1.6) was observed.

### 4.4 Radial profile

**Fig. 21:**
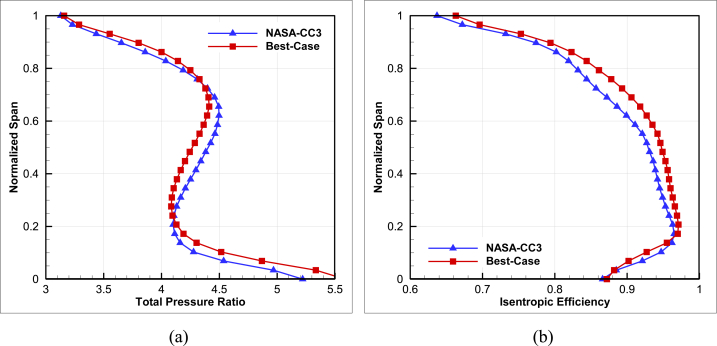
The comparison of radial profile of(a) total pressure ratio vs. normalized span and (b) isentropic efficiency vs. normalized span.

A radial profile distribution has been extracted for the baseline geometry and optimal geometry and shown in Fig. 21.

According to the results, it appears that adding a second splitter in the appropriate location can provide better isentropic efficiency at the design point. Although the optimized compressor's radial total pressure ratio at the outlet varies in some regions with an increase and then a decrease, the overall result is almost equal and close to the total pressure ratio of the baseline compressor.

### 4.5 Circumferential profile

**Fig. 22:**
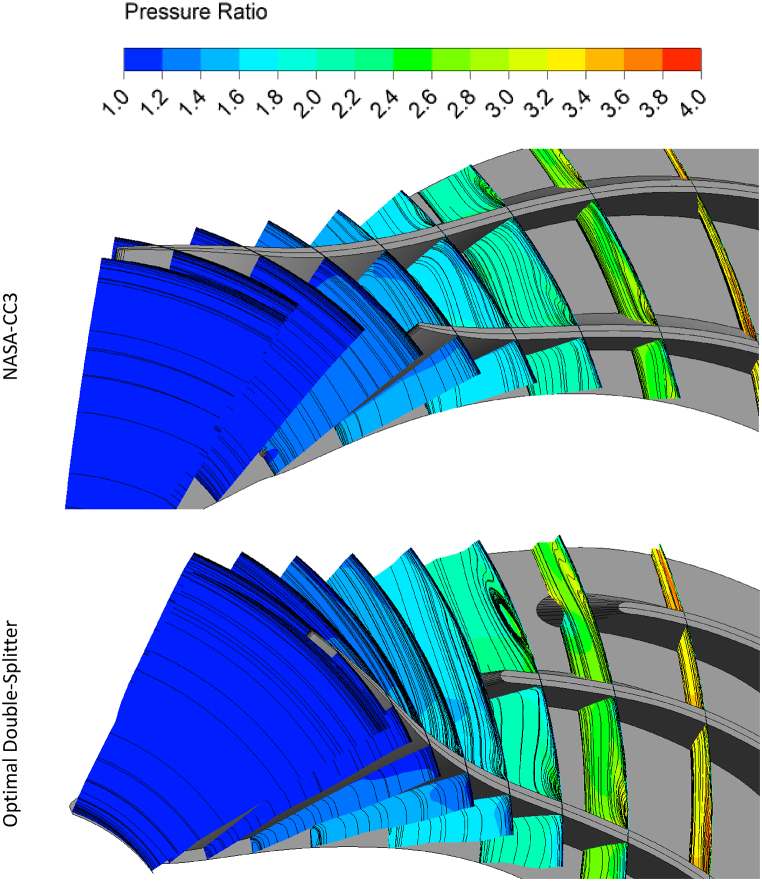
The pressure ratio contours and streamlines of NASA-CC3 and optimal centrifugal compressor from leading edge view.

**Fig. 23:**
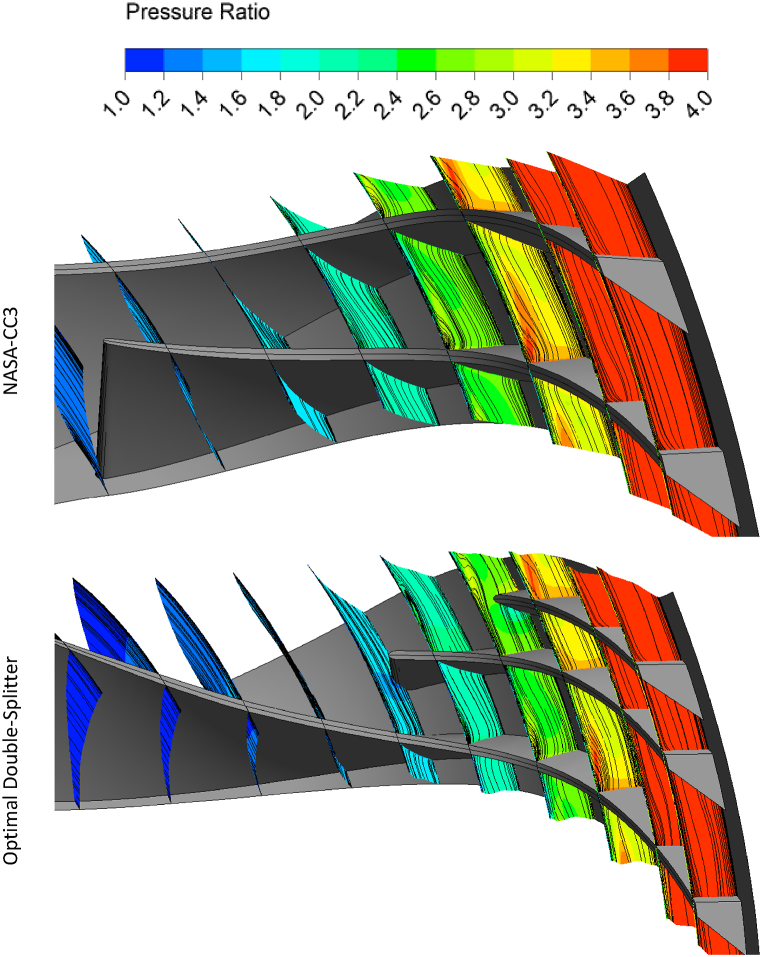
The pressure ratio contours and streamlines of NASA-CC3 and optimal centrifugal compressor from trailing edge view.

Fig. 22, Fig. 23 show the contours of pressure ratio and streamlines in sections of the NASA-CC3 compressor and the optimized compressor from the inlet to the outlet of the impeller. As can be seen, the vortices around the tip of the main blade in the NASA-CC3 compressor and the optimized compressor have not changed much, but it can be said that the vortices around the tip of the splitter blade in the NASA-CC3 compressor have disappeared in the optimized case. Also, in the optimized compressor, before the flow reaches the second splitter blade, the flow vortex is formed, which is destroyed when it collides with this blade, and in fact, it will help to reduce the pressure losses.

As mentioned, the splitter blades reduce the flow separation and make the flow more uniform at the exit, which causes lower pressure losses in the diffuser.

### 4.6 Tip-leakage

**Fig. 24:**
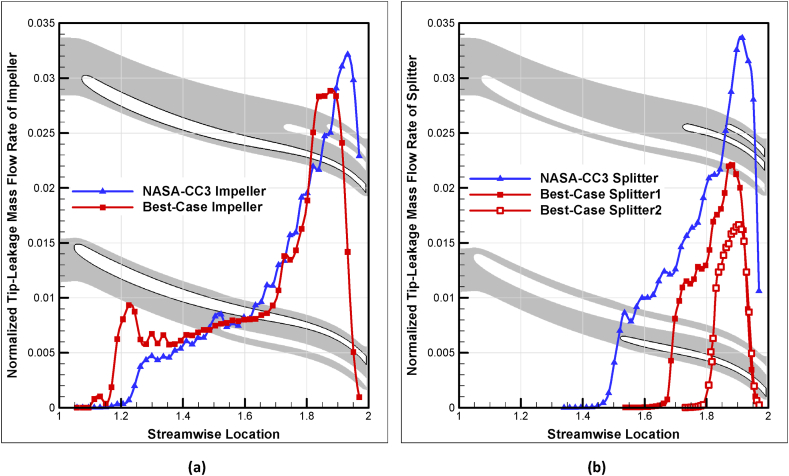
The comparison of tip-leakage mass flow rate-streamwise location of NASA-CC3 and optimal compressor for (a) impeller and (b) splitters.

Another result of the improved performance of the optimized compressor compared to the baseline geometry is a reduction in leakage mass flow at the tip of impeller and splitter blades. The total tip-leakage mass flow of all impeller and splitter blades was calculated using Eqn. (2) and Eqn. (3) and plotted along the streamwise location in Fig. 24.

(2) $${\dot{m}}_{Imp,Leak}=\sum_{Imp}\Delta \dot{m}$$

(3) $${\dot{m}}_{Spl,Leak}=\sum_{Spl}\Delta \dot{m}$$

As can be seen, the mass flow leakage of the optimized impeller is almost always lower than that of the baseline impeller along the entire streamwise locations. This result holds true for the splitters as well, with the mass flow leakage of the first splitter being lower than that of the baseline splitter, and the same being true for the second splitter. It should be noted that the length of the optimized splitter blades is also shorter than that of the baseline splitter blades.

**Fig. 25:**
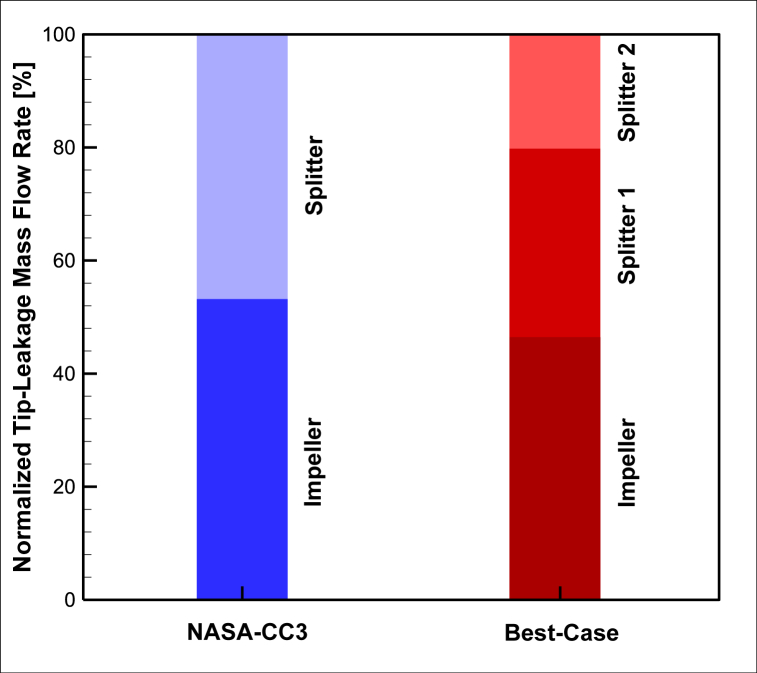
The comparison of normalized tip-leakage mass flow rate of NASA-CC3 and Best-Case.

Finally, the total leakage flow rates of the main blade, first splitter, and second splitter in the optimized baseline compressor geometry are normalized based on the input mass flow rate and displayed in Fig. 25. Table 8 shows the design point leakage flow rates and the normal values of the leakage rates for the impeller and splitters of the two compressors, NASA-CC3 and Best-Case.

**Table 8:** The comparison of normalized tip-leakage mass flow rate.

Case	Impeller [%]	Splitter 1 [%]	Splitter 2 [%]
NASA-CC3	53.25	46.75	–
Best-Case	47.55	31.08	21.37

The impeller leakage flow rate in the optimized compressor geometry is lower than that of the baseline compressor geometry, while the impellers themselves remained unchanged. Similarly, the first splitter leakage flow rate in the optimized compressor geometry is lower than that of the baseline compressor geometry, with only changes in its length and position.

The total tip-leakage flow rate of the impeller and splitter in the baseline compressor geometry is equal to 249 [gr/s], while the total leakage flow rate of the impeller, first splitter, and second splitter in the optimized compressor geometry is equal to 225 [gr/s]. This indicates that the optimized compressor operates with lower leakage flow rates and improved performance.

## 5 Conclusions

In this study, the results of the NASA-CC3 centrifugal compressor with a splitter blade were first validated and the flow field inside this compressor was simulated. Then, in order to study the effect of double-splitter blades in centrifugal compressors, parameterized geometry was used for the design of the second splitter blade, based on the main blade configuration.

Eight optimization variables were selected. Design of Experiments analysis was studied on performance parameters, and the geometry was optimized to increase the on-design efficiency of the compressor while maintaining the massflow rate and pressure ratio.

Due to the computational costs of the optimization process, artificial neural networks were used as response surface functions, trained with data from the Taguchi database and validated. The focus has been on maximizing on-design efficiency.

The results of this optimization process show that while maintaining a flow rate of 4.54 kg/s and a total pressure ratio of 3.88, the efficiency has increased from 83.05% to 84.11%, showing a 1.06% increase. After the optimization process, the performance curve of the optimized compressor was compared with the NASA-CC3 compressor. Isentropic efficiency has increased at all performance points, and it can be stated that the pressure ratio has not changed significantly.

## CRediT authorship contribution statement

**Mohammadjavad Tasharrofi:** Writing – original draft, Visualization, Validation, Software, Methodology, Investigation, Formal analysis, Data curation. **Mojtaba Heidarian Shahri:** Writing – review & editing, Writing – original draft, Visualization, Validation, Software, Methodology, Investigation, Formal analysis, Data curation. **Ali Madadi:** Writing – review & editing, Supervision, Resources, Project administration, Data curation, Conceptualization.

## Data availability statement

The data that support the findings of this study are available on request from the corresponding author.

## Funding

There is no funding for this research.

## Declaration of competing interest

The authors declare that they have no known competing financial interests or personal relationships that could have appeared to influence the work reported in this paper.
